# Islet Cell Associated Autoantibodies and C-Peptide Levels in Patients with Diabetes and Symptoms of Gastroparesis

**DOI:** 10.3389/fendo.2018.00032

**Published:** 2018-02-13

**Authors:** Elias S. Siraj, Carol Homko, Laura A. Wilson, Patrick May, Ajay D. Rao, Jorge Calles, Gianrico Farrugia, William L. Hasler, Kenneth L. Koch, Linda Nguyen, William J. Snape, Thomas L. Abell, Irene Sarosiek, Richard W. McCallum, Pankaj J. Pasricha, John Clarke, James Tonascia, Frank Hamilton, Henry P. Parkman

**Affiliations:** ^1^Division of Endocrinology and Metabolic Disorders, Eastern Virginia Medical School, Norfolk, VA, United States; ^2^Lewis Katz School of Medicine, Temple University, Philadelphia, PA, United States; ^3^Johns Hopkins University, Baltimore, MD, United States; ^4^Case Western Reserve University, Cleveland, OH, United States; ^5^Mayo Clinic, Rochester, NY, United States; ^6^University of Michigan, Ann Arbor, MI, United States; ^7^Wake Forest University, Winston-Salem, NC, United States; ^8^Stanford University, Palo Alto, CA, United States; ^9^California Pacific Medical Center, San Francisco, CA, United States; ^10^University of Louisville, Louisville, KY, United States; ^11^Texas Tech University Health Science Center, El Paso, TX, United States; ^12^National Institute of Diabetes and Digestive and Kidney Diseases, Bethesda, MD, United States

**Keywords:** GAD, GAD65, GAD65 antibodies, islet cell antibodies, C-peptide, gastroparesis, diabetic gastroparesis, gastric emptying

## Abstract

**Introduction:**

Individuals with diabetes are at increased risk for complications, including gastroparesis. Type 1 diabetes mellitus (T1DM) is an autoimmune disorder resulting in decreased beta-cell function. Glutamic acid decarboxylase-65 antibody (GADA) is the most commonly used test to assess autoimmunity while C-peptide level is used to assess beta-cell function. Patients with type 2 diabetes mellitus (T2DM), who are GADA positive, are labeled latent autoimmune diabetes in adults (LADA).

**Objective:**

To characterize patients with T1 and T2DM who have symptoms of gastroparesis using GADA and C-peptide levels and to look for association with the presence of gastroparesis and its symptom severity.

**Design:**

113 T1DM and 90 T2DM patients with symptoms suggestive of gastroparesis were studied. Symptom severity was assessed using Gastroparesis Cardinal Symptom Index (GCSI). Serum samples were analyzed for GADA and C-peptide.

**Results:**

Delayed gastric emptying was present in 91 (81%) of T1DM and 60 (67%) of T2DM patients (*p* = 0.04). GADA was present in 13% of T2DM subjects [10% in delayed gastric emptying and 20% in normal gastric emptying (*p* = 0.2)]. Gastric retention and GCSI scores were mostly similar in GADA positive and negative T2DM patients. GADA was present in 45% of T1DM subjects [46% in delayed gastric emptying and 41% in normal gastric emptying (*p* = 0.81)]. Low C-peptide levels were seen in 79% T1DM patients and 8% T2DM. All seven T2DM patients with low C-peptide were taking insulin compared to 52% of T2DM with normal C-peptide.

**Conclusion:**

GADA was present in 13% while low C-peptide was seen in 8% of our T2DM patients with symptoms of gastroparesis. Neither did correlate with degree of delayed gastric emptying or symptom severity.

**ClinicalTrials.gov Identifier:**

NCT01696747.

## Introduction

Type 1 diabetes mellitus (T1DM) is an autoimmune disorder (with evidence of autoantibodies) and decreased beta-cell function (measured using C-peptide levels), whereas Type 2 diabetes (T2DM) results from a combination of insulin resistance and diminished beta-cell function. However, some patients with T2DM are found to have positive autoantibody profile [often positive glutamic acid decarboxylase-65 antibody (GADA)] suggesting they may have latent autoimmune diabetes in adults (LADA) (1). In studies specific to North America, LADA has been reported in 3–20% of patients initially thought to have T2DM (2, 3).

The presence of LADA in patients clinically considered to have T2DM at diagnosis is found to be associated with a progression to beta-cell failure requiring insulin within few years (4). Individuals with LADA have worse glycemic control than patients with T2DM (5). In addition, it has been reported that LADA patients may have a higher prevalence of complications, particularly retinopathy and nephropathy than T2DM (4).

Gastroparesis is another complication of long-standing diabetes characterized by delayed gastric emptying. Approximately 25–55% of T1DM develop gastroparesis (6). However, gastroparesis is being increasingly diagnosed in type 2 diabetes (T2DM) patients as well with prevalence rate of about 30% (6). A recent study indicated that patients with generalized autoimmune dysautonomia may also present with gastroparesis. Immune dysfunction in such patients can be evaluated using antibodies to glutamic acid decarboxylase (GADA) (7). It is, therefore, interesting to look if presence of GADA in both T1 and T2DM is associated with the presence and severity of gastroparesis.

The aim of this study was to characterize patients with diabetes who have symptoms of gastroparesis using GADA and C-peptide levels to help determine if these correlate with delayed gastric emptying and symptoms, better than the clinical classification of T1DM and T2DM. We also wanted to test the hypothesis that patients with T2DM who are GADA positive are more likely to develop gastroparesis. We are hypothesizing that patients with an autoimmune form of diabetes, whether T1DM or T2DM, may be at a higher risk of developing gastroparesis.

## Materials and Methods

This study is a secondary analysis of data from the Gastroparesis Clinical Research Consortium (GpCRC) Registry (8, 9). The NIDDK GpCRC has a large number of carefully evaluated patients with diabetes and gastroparesis, as well as a number of patients with diabetes with symptoms of gastroparesis but normal gastric emptying. The GpCRC Gastroparesis Registry (GpR) was established in 2006 as an observational study to investigate the etiology, epidemiology, and degree of morbidity with gastroparesis. The second NIDDK GpR (GpR2) was started in 2013, collecting more physiologic testing. Classification of type of diabetes for the registry was obtained from the patient’s medical record and/or in some cases by patient self-report.

The registry collected extensive clinical data on patients in order to fully characterize the features of their gastroparesis. These include a complete medical history, physical examination, gastric emptying scintigraphy (GES), validated symptom questionnaires including Patient Assessment of Upper Gastrointestinal Disorders Symptoms Severity Index (PAGI-SYM) (10) and laboratory tests, including glucose and glycosylated hemoglobin levels. The history asked about the use of insulin and the presence of peripheral neuropathy. Fasting serum and plasma had been stored and were utilized to assess GADA and C-peptide levels for this study.

### Laboratory Analysis

Serum samples were analyzed for GADA and C-peptide levels. The assays were performed through Quest Diagnostics Research Laboratory. GADA levels were measured using a radiobinding assay. The reference value for GADA was ≤1.0 U/ml. C-peptide levels were measured using an immunoassay. The reference range was 0.80–3.10 ng/ml.

### Patient Assessment of GI Symptoms (PAGI-SYM)

The PAGI-SYM questionnaire assessed 24 symptoms of gastroparesis, dyspepsia, gastroesophageal reflux disease with the severity over the last 2 weeks rated by the patient as none = 0 to very severe = 5 (0 = none, 1 = very mild, 2 = mild, 3 = moderate, 4 = severe, 5 = very severe) (10). This PAGI-SYM contains the nine symptoms comprising the Gastroparesis Cardinal Symptom Index (GCSI) (11). Patients were also asked about their most prominent symptom.

### Gastric Emptying Scintigraphy

Gastric emptying scintigraphy was performed using a low-fat, egg white meal with imaging at 0, 1, 2, and 4 h after meal ingestion (12). This protocol ensured standardized information about gastric emptying across multiple sites. Patients are instructed to stop medications that could affect gastrointestinal motility for 48 h prior to the study and to come to the Nuclear Medicine Section in the morning after fasting overnight, that is, an 8-h fast. Patients with diabetes have their glucose checked at the beginning of the study, with appropriate treatment measures being taken if low blood sugar (hypoglycemia < 60 mg/dl) or high blood sugar (hyperglycemia > 250 mg/dl) is detected. GES is performed using a standard low-fat, Eggbeaters^®^ meal to measure solid emptying. The meal consists of the equivalent of two large eggs radiolabeled with 0.5–1 mCi Tc-99m sulfur colloid served with two pieces of white bread and jelly. Patients are given 120 ml water. Following ingestion of the meal, imaging is performed at 0, 1, 2, and 4 h with the patient upright for measuring gastric emptying of Tc-labeled solids. Gastric emptying is analyzed as percent of radioactivity retained in the stomach over time using the geometric center of the decay-corrected anterior and posterior counts for each time point. Gastric retention of Tc-99m > 60% at 2 h and/or >10% at 4 h is considered delayed gastric emptying of solids (12).

### Statistical Methods

Descriptive statistics (means, SDs, frequencies, and percentages) were used to compare subgroups of gastroparesis patients. Enrollment characteristics such as demographics, medical history, gastroparesis history, diabetes history and treatment, symptom severity were compared by etiology (T1DM vs T2DM). Within the groups of T1 and T2DM, enrollment characteristics were also compared by the subgroups of patients with positive GAD65 vs negative GAD65 and for subgroups of patients with low vs normal C-peptide levels. *p*-Values were determined from Fisher’s exact tests for categorical variables and *t*-tests for continuous variables. All *p*-values are two-sided; values < 0.05 were considered statistically significant. Analyses were performed using methods described in SAS version 9.3 (SAS Institute) or Stata version 13.1 (StataCorp).

## Results

A total of 203 patients with diabetes (113 patients with T1DM and 90 patients with T2DM) having symptoms suggestive of gastroparesis from the NIDDK GpR were assessed in this study.

### Comparing T1DM vs T2DM

Table 1 shows the baseline characteristics of patients by type of diabetes. As expected, T2DM patients were older, had higher BMI, and less often used insulin compared to T1DM patients. At enrollment into the registry, T1DM patients had a longer duration of diabetes and longer duration of gastroparesis than T2DM patients. At enrollment, 108 (95.6%) of T1DM and 50 (55.6%) of T2DM were using insulin. A1c levels were higher in T1DM (8.2 ± 1.8%) than T2DM (7.6 ± 1.8%, *p* = 0.02). GADA were present in 50 (45%) of T1DM and 12 (13%) of T2DM. Low C-peptide levels were seen in 88 (79%) of T1DM patients and 7 (8%) of T2DM.

**Table 1:** Baseline characteristics patients by diabetes type [Type 1 diabetics (T1DM) vs Type 2 diabetics (T2DM)].

Baseline characteristic	Type 1 diabetes (*n* = 113)*N*(%) or mean ± SD	Type 2 Diabetes (*n* = 90)*N*(%) or mean ± SD	*p*-Value
Demographic			
Gender: female	78 (69.0)	66 (73.3)	0.54
Mean age at enrollment	40.3 ± 12.1	52.0 ± 9.9	<0.001
Ethnicity: Hispanic vs other	17 (15.0)	12 (13.3)	0.84
Race			0.83
White	84 (75.7)	70 (78.7)	
Black	21 (18.9)	16 (18.0)	
Other	6 (5.4)	3 (3.4)	
Anthropometric			
BMI (kg/m^2^)	26.9 ± 6.3	33.3 ± 7.8	<0.001
Medical history			
Age at diabetes diagnosis (years)	18.4 ± 11.1	39.6 ± 10.5	<0.001
Duration of diabetes at enrollment (years)	21.9 ± 12.2	12.4 ± 7.6	<0.001
Presence of peripheral neuropathy	53 (46.9)	32 (35.6)	0.12
Use of insulin	108 (95.6)	50 (55.6)	<0.001
Onset of symptoms			0.29
Acute start	55 (48.7)	46 (51.1)	
Insidious	58 (51.3)	42 (46.7)	
Glucose (mg/dl)	174.9 ± 88.0	148.5 ± 71.9	0.02
HbA1c (%)	8.2 ± 1.8	7.6 ± 1.8	0.02
History of gastroparesis			
Age at diagnosis of gastroparesis (years)	34.0 ± 11.3	47.7 ± 10.5	<0.001
Duration of gastroparesis at enrollment (years)	6.7 ± 6.2	4.3 ± 3.7	0.001
Gastric emptying (mean% retained)			
2-h emptying	61.0 ± 26.9	48.4 ± 25.3	<0.001
4-h emptying	37.3 ± 29.4	21.4 ± 21.2	<0.001
Delayed gastric emptying^a^	91 (80.5)	60 (66.7)	0.04
Islet autoantibodies			
GADA > 1.0 U/ml (positive)	50 (44.6)	12 (13.3)	<0.001
C-Peptide, low (≤0.8 ng/ml)	88 (79.3)	7 (7.8)	<0.001

PAGI-SYM symptom severity (0–5)			
Nausea severity	3.4 ± 1.3	3.1 ± 1.3	0.18
Retching severity	2.4 ± 1.7	2.2 ± 1.6	0.43
Vomiting severity	2.6 ± 1.9	2.0 ± 1.8	0.01
Feeling of stomach fullness severity	3.3 ± 1.5	3.6 ± 1.2	0.08
Inability to finish meal severity	2.8 ± 1.6	3.3 ± 1.4	0.05
Excessively full after meal severity	3.4 ± 1.5	3.6 ± 1.2	0.23
Loss of appetite severity	2.8 ± 1.6	2.8 ± 1.5	0.96
Bloating severity	3.0 ± 1.6	3.4 ± 1.5	0.07
Visibly larger stomach severity	2.7 ± 1.7	2.9 ± 1.7	0.27
Upper abdominal pain	2.8 ± 1.8	2.7 ± 1.7	0.58
Upper abdominal discomfort	2.9 ± 1.7	2.9 ± 1.6	0.83
Cardinal symptom index (GCSI) (0–5)	2.9 ± 1.1	3.0 ± 1.0	0.66
Nausea/vomiting GCSI subscale	2.8 ± 1.5	2.4 ± 1.3	0.06
Bloating GCSI subscale	2.8 ± 1.6	3.2 ± 1.6	0.14
Postprandial fullness GCSI subscale	3.1 ± 1.2	3.3 ± 1.1	0.15

*^a^Gastric emptying scintigraphy of >60% at 2 h OR >10% at 4 h*.

*GCSI, Gastroparesis Cardinal Symptom Index*.

Delayed gastric emptying was present in 91 (81%) of T1DM and 60 (67%) of T2DM patients (*p* = 0.04). Total gastroparesis symptoms (GCSI) were similar between T1DM (2.9 ± 1.1) and T2DM (3.0 ± 1.0; *p* = 0.66), though vomiting was more severe in T1DM (2.6 ± 1.9 vs 2.0 ± 1.8; *p* = 0.01) and early satiety marginally more severe in T2DM (3.3 ± 1.4 vs 2.8 ± 1.6; *p* = 0.05).

### Comparing Glutamic Acid Decarboxylase-65 Antibody (GADA) Positive vs GADA Negative Subjects

Table 2 shows comparison of baseline characteristics between GADA positive and GADA negative subgroups within T1DM. In relative terms, whites were overrepresented in the GADA positive group while Blacks were overrepresented in the GADA negative group (*p* = 0.004). Between the two groups, there was no significant difference in duration of gastroparesis symptoms, gastric emptying or GI symptoms. Low C-peptide levels were seen in 43 (88%) of GADA positive and 45 (73%) of GADA negative subjects.

**Table 2:** Baseline characteristics of patients with T1DM by level of GAD-65 Antibodies.

Baseline characteristic	Positive GAD-65 Antibodies (GADA > 1.0) (*n* = 50)*N*(%) or mean ± SD	Negative GAD-65 Antibodies (GADA ≤ 1.0) (*n* = 62)*N*(%) or mean ± SD	*p*-Value
Demographic			
Gender: female	36 (72.0)	41 (66.1)	0.54
Mean age at enrollment	40.6 ± 12.4	40.4 ± 11.8	0.93
Ethnicity: Hispanic vs other	8 (16.0)	9 (14.5)	1.00
Race			0.004
White	45 (90.0)	39 (65.0)	
Black	3 (6.0)	17 (28.3)	
Other	2 (4.0)	4 (6.7)	
Anthropometric			
BMI (kg/m^2^)	26.8 ± 6.5	27.1 ± 6.2	0.79
Medical history			
Diabetes			
Age at diabetes diagnosis (years)	19.9 ± 12.3	17.5 ± 9.9	0.26
Duration of diabetes at enrollment (years)	20.7 ± 11.9	23.0 ± 12.5	0.34
Presence of peripheral neuropathy	24 (48.0)	28 (45.2)	0.85
Use of insulin	47 (94.0)	60 (96.8)	0.65
Infectious prodrome	7 (14.0)	9 (14.5)	1.00
Onset of symptoms			0.26
Acute start	21 (42.0)	33 (53.2)	
Insidious	29 (58.0)	29 (46.8)	
Glucose (mg/dl)	179.5 ± 96.8	171.7 ± 81.6	0.65
HbA1c (%)	8.3 ± 2.0	8.1 ± 1.8	0.43
History of gastroparesis			
Age at diagnosis of gastroparesis (years)	33.7 ± 12.1	34.4 ± 10.7	0.74
Duration of gastroparesis at enrollment (years)	7.2 ± 7.3	6.3 ± 5.2	0.43
Gastric emptying (mean% retained)			
2-h emptying	61.4 ± 25.6	60.2 ± 28.1	0.81
4-h emptying	36.2 ± 28.3	37.7 ± 30.5	0.79
Delayed gastric emptying^a^	41 (82.0)	49 (79.0)	0.81
C-peptide, low (≤0.8 ng/ml)	43 (87.8)	45 (72.6)	0.06

PAGI-SYM symptom severity (0–5)			
Nausea severity	3.3 ± 1.3	3.5 ± 1.4	0.52
Retching severity	2.3 ± 1.7	2.4 ± 1.7	0.76
Vomiting severity	2.6 ± 1.9	2.7 ± 1.8	0.73
Feeling of stomach fullness severity	3.3 ± 1.5	3.2 ± 1.5	0.69
Inability to finish meal severity	2.8 ± 1.5	2.9 ± 1.7	0.71
Excessively full after meal severity	3.4 ± 1.5	3.5 ± 1.5	0.75
Loss of appetite severity	3.0 ± 1.6	2.7 ± 1.6	0.37
Bloating severity	3.0 ± 1.5	3.1 ± 1.6	0.73
Visibly larger stomach severity	2.6 ± 1.7	2.7 ± 1.8	0.67
Upper abdominal pain	2.7 ± 1.8	2.9 ± 1.8	0.66
Upper abdominal discomfort	2.9 ± 1.7	3.0 ± 1.7	0.89
Cardinal symptom index (GCSI) (0–5)	2.9 ± 1.1	3.0 ± 1.0	0.73
Nausea/vomiting GCSI subscale	2.8 ± 1.5	2.9 ± 1.5	0.65
Bloating GCSI subscale	2.8 ± 1.5	2.9 ± 1.6	0.69
Postprandial fullness GCSI subscale	3.1 ± 1.3	3.1 ± 1.2	0.86

*^a^Gastric emptying scintigraphy of >60% at 2 h OR >10% at 4 h*.

*GCSI, Gastroparesis Cardinal Symptom Index*.

Table 3 shows comparison of baseline characteristics between GADA positive and GADA negative subgroups within T2DM. GADA positive subjects with T2DM, compared to GADA negatives, had a longer duration of gastroparesis (7.1 ± 6.7 vs 3.8 ± 2.9 years; *p* = 0.004), marginally lower prevalence of peripheral neuropathy (8.3 vs 39.7%; *p* = 0.05), and were more likely to be Hispanic (42 vs 9%; *p* = 0.009). Regarding symptoms, upper abdominal pain was higher in GADA positive subjects (3.7 ± 1.6 vs 2.5 ± 1.7; *p* = 0.03) but the rest of the symptoms and gastric emptying results were similar.

**Table 3:** Baseline characteristics of patients with T2DM by level of GAD-65 Antibodies.

Baseline characteristic	Positive GAD-65 Antibodies (GADA > 1.0) (*n* = 12)*N*(%) or mean ± SD	Negative GAD-65 Antibodies (GADA ≤ 1.0) (*n* = 78)*N*(%) or mean ± SD	*p-*Value
Demographic			
Gender: female	8 (66.7)	58 (74.4)	0.73
Mean age at enrollment	51.2 ± 12.3	52.1 ± 9.6	0.76
Ethnicity: Hispanic vs other	5 (41.7)	7 (9.0)	0.009
Race			0.32
White	8 (66.7)	62 (80.5)	
Black	4 (33.3)	12 (15.6)	
Other	0 (–)	3 (3.9)	
Anthropometric			
BMI (kg/m^2^)	31.9 ± 9.3	33.5 ± 7.5	0.51
Medical history			
Diabetes			
Age at diabetes diagnosis (years)	38.9 ± 11.0	39.7 ± 10.5	0.80
Duration of diabetes at enrollment (years)	12.3 ± 9.3	12.4 ± 7.3	0.96
Presence of peripheral neuropathy	1 (8.3)	31 (39.7)	0.05
Use of insulin	5 (41.7)	45 (57.7)	0.36
Onset of symptoms			0.28
Acute start	5 (41.7)	41 (52.6)	
Insidious	6 (50.0)	36 (46.2)	
Glucose (mg/dl)	148.8 ± 80.4	148.4 ± 71.1	0.99
HbA1c (%)	7.3 ± 1.6	7.6 ± 1.8	0.56
History of gastroparesis			
Age at diagnosis of gastroparesis (years)	44.1 ± 12.5	48.3 ± 10.1	0.19
Duration of gastroparesis at enrollment (years)	7.1 ± 6.7	3.8 ± 2.9	0.004
Gastric emptying (mean% retained)			
2-h emptying	44.5 ± 21.3	49.0 ± 26.0	0.56
4-h emptying	13.8 ± 11.6	22.5 ± 22.1	0.19
Delayed gastric emptying^a^	6 (50.0)	54 (69.2)	0.20
C-Peptide, low (≤0.8 ng/ml)	1 (8.3)	6 (7.7)	1.00

PAGI-SYM symptom severity (0–5)			
Nausea severity	3.2 ± 1.2	3.1 ± 1.4	0.93
Retching severity	1.9 ± 1.7	2.2 ± 1.6	0.56
Vomiting severity	2.1 ± 2.0	1.9 ± 1.7	0.81
Feeling of stomach fullness severity	3.9 ± 0.8	3.5 ± 1.3	0.32
Inability to finish meal severity	3.3 ± 1.5	3.3 ± 1.4	0.87
Excessively full after meal severity	3.7 ± 1.1	3.6 ± 1.2	0.92
Loss of appetite severity	2.7 ± 1.5	2.8 ± 1.5	0.72
Bloating severity	3.9 ± 0.9	3.3 ± 1.6	0.19
Visibly larger stomach severity	3.6 ± 1.3	2.8 ± 1.8	0.17
Upper abdominal pain	3.7 ± 1.6	2.5 ± 1.7	0.03
Upper abdominal discomfort	3.7 ± 1.5	2.8 ± 1.6	0.08
Cardinal symptom index (GCSI) (0–5)	3.2 ± 0.9	2.9 ± 1.1	0.48
Nausea/vomiting GCSI subscale	2.4 ± 1.4	2.4 ± 1.3	0.93
Bloating GCSI subscale	3.8 ± 1.0	3.1 ± 1.6	0.16
Postprandial fullness GCSI subscale	3.4 ± 1.1	3.3 ± 1.2	0.90

*^a^Gastric emptying scintigraphy (GES) of >60% at 2 h OR >10% at 4 h*.

### Comparison by Gastric Emptying Results within T2DM

Table 4 shows comparison of baseline characteristics between those with normal gastric emptying vs those with delayed gastric emptying subgroups within T2DM. Females were marginally overrepresented in the delayed gastric emptying group (*p* = 0.08). GADA was present in 13% of T2DM subjects [10% in delayed gastric emptying and 20% in normal gastric emptying (*p* = 0.2)]. There was no significant difference in GADA positivity or the frequency of low C-peptide between the two groups.

**Table 4:** Baseline characteristics of patients with Type 2 Diabetes (T2DM) by presence of delayed *gastric emptying*.

Baseline characteristic	Normal emptying (*n* = 30)*N*(%) or mean ± SD	Delayed emptying^a^ (*n* = 60)*N*(%) or mean ± SD	*p*-Value
Demographic			
Gender: female	18 (60.0)	48 (80.0)	0.08
Mean age at enrollment	50.5 ± 8.5	52.8 ± 10.6	0.30
Ethnicity: Hispanic vs other	5 (16.7)	7 (11.7)	0.53
Race			0.43
White	23 (76.7)	47 (79.7)	
Black	7 (23.3)	9 (15.3)	
Other	0 (0)	3 (5.1)	
Anthropometric			
BMI (kg/m^2^)	33.6 ± 8.3	33.2 ± 7.5	0.81
Medical history			
Diabetes			
Age at diabetes diagnosis (years)	40.0 ± 8.7	39.4 ± 11.4	0.81
Duration of diabetes at enrollment (years)	10.5 ± 6.0	13.3 ± 8.1	0.09
Presence of peripheral neuropathy	10 (33.3)	22 (36.7)	0.82
Use of insulin	18 (60.0)	32 (53.3)	0.65
Onset of symptoms			0.24
Acute start	19 (63.3)	27 (45.0)	
Insidious	11 (36.7)	31 (51.7)	
Other	0 (-)	2 (3.3)	
Glucose (mg/dl)	144.3 ± 48.9	150.5 ± 81.3	0.70
HbA1c (%)	7.6 ± 1.5	7.6 ± 1.9	0.93
History of gastroparesis			
Age at diagnosis of gastroparesis (years)	45.3 ± 9.5	49.0 ± 10.8	0.12
Duration of gastroparesis at enrollment (years)	5.2 ± 4.9	3.8 ± 2.9	0.11
Gastric emptying (mean% retained)			
2-h emptying	26.1 ± 18.1	59.6 ± 20.7	<0.001
4-h emptying	3.6 ± 3.0	30.3 ± 20.8	<0.001
GADA positive (>1.0 U/ml)	6 (20.0)	6 (10.0)	0.20
C-Peptide, low (≤0.8 ng/ml)	1 (3.3)	6 (10.0)	0.42

PAGI-SYM symptom severity (0–5)			
Nausea severity	3.1 ± 1.3	3.1 ± 1.4	1.00
Retching severity	1.8 ± 1.6	2.3 ± 1.6	0.16
Vomiting severity	1.5 ± 1.6	2.2 ± 1.8	0.07
Feeling of stomach fullness severity	3.6 ± 1.1	3.6 ± 1.3	0.81
Inability to finish meal severity	3.2 ± 1.6	3.4 ± 1.3	0.71
Excessively full after meal severity	3.7 ± 1.1	3.6 ± 1.3	0.59
Loss of appetite severity	2.8 ± 1.6	2.8 ± 1.4	0.92
Bloating severity	3.4 ± 1.4	3.4 ± 1.6	0.92
Visibly larger stomach severity	3.0 ± 1.7	2.9 ± 1.8	0.80
Upper abdominal pain	2.6 ± 1.8	2.7 ± 1.7	0.76
Upper abdominal discomfort	2.8 ± 1.6	3.0 ± 1.6	0.61
Cardinal symptom index [Gastroparesis Cardinal Symptom Index (GCSI)] (0–5)	2.9 ± 1.0	3.0 ± 1.1	0.64
Nausea/vomiting GCSI subscale	2.2 ± 1.2	2.6 ± 1.4	0.18
Bloating GCSI subscale	3.2 ± 1.5	3.2 ± 1.6	0.92
Postprandial fullness GCSI subscale	3.3 ± 1.2	3.3 ± 1.1	0.88

*^a^Delayed gastric emptying defined as having gastric emptying scintigraphy of >60% at 2 h OR >10% at 4 h*.

### Comparison by Gastric Emptying Results within T1DM

Glutamic acid decarboxylase-65 antibody was present in 45% of T1DM subjects [46% in delayed gastric emptying and 41% in normal gastric emptying (*p* = 0.81)]. Low C-peptide levels were seen in 79% of T1DM [79% in delayed gastric emptying and 82% in normal gastric emptying (*p* = 1.00)]. The full comparison is shown on Table 5.

**Table 5:** Baseline characteristics of patients with Type 1 Diabetes (T1DM) by presence of delayed *gastric emptying*.

Baseline characteristic	Normal emptying (*n* = 22)*N*(%) or Mean ± SD	Delayed Emptying^a^ (*n* = 91)*N*(%) or Mean ± SD	*p-*Value
Demographic			
Gender: female	18 (81.8)	60 (65.9)	0.20
Mean age at enrollment	44.3 ± 14.0	39.4 ± 11.5	0.08
Ethnicity: Hispanic vs other	4 (18.2)	13 (14.3)	0.74
Race			0.82
White	18 (81.8)	66 (74.2)	
Black	3 (13.6)	18 (20.2)	
Other	1 (4.6)	5 (5.6)	
Anthropometric			
BMI (kg/m^2^)	29.1 ± 6.8	26.3 ± 6.1	0.06
Medical history			
Diabetes			
Age at diabetes diagnosis (years)	19.2 ± 13.4	18.3 ± 10.5	0.73
Duration of diabetes at enrollment (years)	25.2 ± 15.0	21.1 ± 11.4	0.16
Presence of peripheral neuropathy	13 (59.1)	40 (44.0)	0.24
Use of insulin	22 (100.0)	86 (94.5)	0.58
Onset of symptoms			0.03
Acute start	6 (27.3)	49 (53.9)	
Insidious	16 (72.7)	42 (46.2)	
Glucose (mg/dl)	187.1 ± 108.9	172.0 ± 82.6	0.47
HbA1c (%)	8.3 ± 1.6	8.2 ± 1.9	0.77
History of gastroparesis			
Age at diagnosis of gastroparesis (years)	36.4 ± 15.1	33.4 ± 10.3	0.27
Duration of gastroparesis at enrollment (years)	8.4 ± 7.6	6.3 ± 5.8	0.17
Gastric emptying (mean% retained)			
2-h emptying	23.1 ± 16.2	70.1 ± 20.1	<0.001
4-h emptying	4.1 ± 2.8	45.3 ± 27.2	<0.001
GADA positive (>1.0 U/ml)	9 (40.9)	41 (45.6)	0.81
C-Peptide, low (≤0.8 ng/ml)	18 (81.8)	70 (78.7)	1.00

PAGI-SYM symptom severity (0–5)			
Nausea severity	3.3 ± 1.6	3.4 ± 1.3	0.65
Retching severity	2.1 ± 1.8	2.4 ± 1.7	0.51
Vomiting severity	2.5 ± 2.0	2.7 ± 1.8	0.56
Feeling of stomach fullness severity	3.6 ± 1.3	3.2 ± 1.5	0.17
Inability to finish meal severity	3.0 ± 1.4	2.8 ± 1.6	0.60
Excessively full after meal severity	3.6 ± 1.5	3.3 ± 1.5	0.41
Loss of appetite severity	2.9 ± 1.6	2.8 ± 1.6	0.78
Bloating severity	3.5 ± 1.5	2.9 ± 1.6	0.07
Visibly larger stomach severity	3.2 ± 1.7	2.5 ± 1.7	0.09
Upper abdominal pain	2.8 ± 1.6	2.8 ± 1.8	0.99
Upper abdominal discomfort	3.0 ± 1.6	3.0 ± 1.7	0.93
Cardinal symptom index [Gastroparesis Cardinal Symptom Index (GCSI)] (0–5)	3.1 ± 1.2	2.9 ± 1.1	0.34
Nausea/vomiting GCSI subscale	2.6 ± 1.7	2.8 ± 1.5	0.53
Bloating GCSI subscale	3.4 ± 1.5	2.7 ± 1.6	0.07
Postprandial fullness GCSI subscale	3.3 ± 1.3	3.0 ± 1.2	0.36

*^a^Delayed gastric emptying defined as having gastric emptying scintigraphy of >60% at 2 h OR >10% at 4 h*.

### Comparing Low C-Peptide vs Normal/High C-Peptide Subjects

Table 6 shows comparison of baseline characteristics between low C-peptide and normal/high C-peptide subgroups within T1DM. Of all T1DM patients, 88 (79%) had low C-peptide levels while the rest 23 (21%) had normal/high C-peptide levels, indicating the limitations of clinical classification. All of the 88 subjects with low C-peptide levels (100%) were taking insulin, compared to only 18 (78%) of those with normal/high C-peptide (*p* < 0.001). GADA positivity was marginally higher in the low C-peptide group (*p* = 0.06). There were no significant differences between the two groups in gastric emptying as well as in symptoms.

**Table 6:** Baseline characteristics of patients with Type 1 Diabetes (T1DM) by level of C-Peptide.

Baseline characteristic	Low C-peptide (≤0.8 ng/ml)^a^ (*n* = 88)*N*(%) or Mean ± SD	Normal/high C-peptide (>0.8 ng/ml)^a^ (*n* = 23)*N*(%) or Mean ± SD	*p*-Value
Demographic			
Gender: female	63 (71.6)	13 (56.5)	0.21
Mean age at enrollment	40.0 ± 12.3	42.6 ± 11.0	0.36
Ethnicity: Hispanic vs other	8 (9.1)	8 (34.8)	0.005
Race			0.39
White	69 (78.4)	14 (66.7)	
Black	15 (17.1)	5 (23.8)	
Other	4 (4.6)	2 (9.5)	
Anthropometric			
BMI (kg/m^2^)	26.9 ± 6.5	27.2 ± 6.0	0.87
Medical history			
Diabetes			
Age at diabetes diagnosis (years)	17.2 ± 10.5	24.0 ± 11.9	0.008
Duration of diabetes at enrollment (years)	22.8 ± 12.6	18.6 ± 10.7	0.15
Presence of peripheral neuropathy	40 (45.5)	12 (52.2)	0.64
Use of insulin	88 (100.0)	18 (78.3)	<0.001
Onset of symptoms			1.00
Acute start	42 (47.7)	11 (47.8)	
Insidious	46 (52.3)	12 (52.2)	
Glucose (mg/dl)	172.5 ± 83.6	174.9 ± 94.8	0.90
HbA1c (%)	8.3 ± 1.6	7.5 ± 2.4	0.04
History of gastroparesis			
Age at diagnosis of gastroparesis (years)	33.4 ± 11.5	37.3 ± 10.1	0.14
Duration of gastroparesis at enrollment (years)	7.0 ± 6.2	5.4 ± 6.4	0.27
Gastric emptying (mean% retained)			
2-h emptying	60.9 ± 26.5	60.4 ± 29.5	0.93
4-h emptying	35.6 ± 29.5	42.3 ± 29.8	0.33
Delayed gastric emptying^b^	70 (79.6)	19 (82.6)	1.00
GADA positive (>1.0 U/ml)	43 (48.9)	6 (26.1)	0.06

PAGI-SYM symptom severity (0–5)			
Nausea severity	3.4 ± 1.3	3.4 ± 1.5	0.93
Retching severity	2.3 ± 1.7	2.7 ± 1.8	0.28
Vomiting severity	2.6 ± 1.9	3.1 ± 1.6	0.25
Feeling of stomach fullness severity	3.2 ± 1.5	3.3 ± 1.3	0.85
Inability to finish meal severity	2.8 ± 1.6	3.2 ± 1.4	0.25
Excessively full after meal severity	3.4 ± 1.6	3.6 ± 1.0	0.57
Loss of appetite severity	2.8 ± 1.7	3.0 ± 1.4	0.57
Bloating severity	3.0 ± 1.6	2.9 ± 1.5	0.64
Visibly larger stomach severity	2.6 ± 1.8	2.7 ± 1.6	0.82
Upper abdominal pain	2.8 ± 1.8	3.0 ± 1.8	0.55
Upper abdominal discomfort	3.0 ± 1.7	3.0 ± 1.8	0.98
Cardinal symptom index [Gastroparesis Cardinal Symptom Index (GCSI)] (0–5)	2.9 ± 1.1	3.0 ± 0.9	0.52
Nausea/vomiting GCSI subscale	2.8 ± 1.5	3.1 ± 1.5	0.39
Bloating GCSI subscale	2.8 ± 1.7	2.8 ± 1.4	0.91
Postprandial fullness GCSI subscale	3.0 ± 1.3	3.3 ± 0.9	0.44

*^a^Negative C-Peptide defined as c-peptide levels ≤0.8 ng/ml*.

*^b^Gastric emptying scintigraphy of >60% at 2 h OR >10% at 4 h*.

*GCSI, Gastroparesis Cardinal Symptom Index*.

We compared the baseline characteristics between low C-peptide and normal/high C-peptide subgroups within T2DM. Of all T2DM patients, 7 (7.7%) had low C-peptide levels while the rest 83 (92.3%) had normal/high C-peptide levels. All of the seven subjects with low C-peptide levels (100%) were taking insulin, compared to only 43 (51%) of those with normal/high C-peptide. GADA positivity was not significantly different between the two groups. There were no significant differences between the two groups in gastric emptying, but the severity of bloating and visibly larger stomach was greater in those with normal/high C-peptide levels. The significance of this data is limited due to the small number of patients with T2DM who have low C-peptide levels.

## Discussion

The classification between T1DM and T2DM is generally done using clinical criteria. Occasionally, GADA and C-peptide levels are measured to help differentiate between the two. Generally, GADA positivity and low C-peptide levels are considered as indicators of the presence of T1DM. GADA positivity, which could be seen in >80% of T1DM patients at the time of diagnosis, tends to decline over time. On the other side, a minority of T2DM patients have been recognized to have GADA positivity and low C-peptide levels. The name latent autoimmune diabetes in adults (LADA) has been used to denote those subjects, who also tend to demonstrate some phenotypic features of T1DM.

About 21% of T1DM subjects did not have low C-peptide levels and about 4% were not on insulin. It is possible that some of those subjects may have been wrongly classified based on clinical parameters. On the other hand, only 45% of T1DM subjects were GADA positive, which is acceptable given the fact that the duration of diabetes was about 21 years, leading to lower positivity.

Our analysis showed that T1DM subjects had longer duration of symptoms and more prevalent delayed gastric emptying than T2DM, but total symptom scores were mostly similar. The only significant difference in symptoms was seen in vomiting, which was more prevalent in T1DM. These data support a prior publication of ours looking at the baseline characteristics and course of T1DM vs T2DM patients (13).

Within T1DM, there was no difference between GADA positive and GADA negative subjects in duration of symptoms, symptom scores and prevalence of delayed gastric emptying.

In regard to T2DM, about 13% were GADA positive and 8% had low C-peptide levels. It is conceivable that those may belong to the category of LADA.

Within T2DM, GADA positives had longer duration of symptoms, but similar prevalence of gastric emptying. They also have similar symptom scores with the exception of upper abdominal pain which was higher in the GADA positive group. Also within this group, GADA positivity and prevalence of low C-peptide were similar when compared between those with delayed gastric emptying and those without.

Within T2DM, comparison between those with low C-peptide vs normal/high C-peptide showed similar duration of symptoms and prevalence of delayed gastric emptying. But, there was more prevalent severity of bloating and visibly larger stomach in those with normal/high C-peptide. These results should be interpreted with caution because of the low number of patients with T2DM who have low C-peptide.

The reported occurrence of LADA in T2DM has been varied. The prevalence of autoantibodies has been reported as anywhere from 3 to 31% with rates varying greatly by geographic area. In studies specific to North America, LADA has been reported in 3.4, 4.7, 5.9, 16, and 20% of patients (2, 3). In the present study, we found that 13% of subjects with phenotypic T2DM were GADA positive. This rate falls within the mid-range of previously reported rates and demonstrates that LADA is not as rare as once thought. In our patients, the rate of gastroparesis was not different between GAD positive and GAD negative phenotypic T2DM patients and we were unable to support our hypothesis that GADA positivity in phenotypically T2DM patients may predispose to gastroparesis.

The diagnosis of LADA mainly relies on seropositivity of antibodies (14). There are three main serum autoantibodies reflecting humoral immunity of LADA: anti-GAD65 antibody (GADA), insulinoma 2-associated antibodies (IA-2A), and insulin autoantibody (IAA). Others include ICA (islet cell cytoplasmic autoantibodies), and zinc transporter 8 autoantibody (ZnT8A). GADA, which is a specific antibody against GAD-65 is recognized as the most sensitive immune parameter for the diagnosis of T1DM and LADA, because it appears early and remains for a long duration in serum. The assay of GAD-65 antibody is the most standardized of all the autoantibodies. Furthermore, in our preliminary work conducted in subjects with gastroparesis and either T1DM or T2DM, no patients were positive for ICA or IAA (15). For these reasons, we limited our evaluation to the anti-GAD65 antibody in the current study.

Characteristics of GADA positive patients with phenotypically type 2 diabetes have been previously reported by several investigators. Arikan and colleagues found that GADA positive patients were significantly younger at age of diabetes onset, had a lower BMI, and lower serum C-peptide levels than patients who were GADA negative with T2DM (4). Hawa and colleagues also found that GADA positive patients had a significantly lower mean age of onset of diabetes as well as lower BMI in their cohort of European subjects (3). In contrast, Zinman and colleagues did find differences in adiposity between GADA positive and negative subjects with type 2 diabetes in the ADOPT study but did find lesser degrees of insulin resistance and a lower probability of the metabolic syndrome (2). In our population, GADA positive patients tended to be younger at diabetes diagnosis and had a lower BMI, even though not statistically significant. In addition, GADA positive patients had a significantly longer duration of gastroparesis at enrollment (7.1 ± 6.7 vs 3.8 ± 2.9 years; *p* = 0.004) than did negative subjects. When comparing phenotypic T2DM subjects, GADA positive patients, had a longer duration of gastroparesis, lower prevalence of peripheral neuropathy, and were more likely to be Hispanic than GADA negative subjects.

In addition to phenotypic characteristics, some investigators have examined the occurrence of the long-term complications among patients who are GADA positive vs negative. In 2003, Arkian et al. reported a higher rate of retinopathy and nephropathy among their GADA positive patients; however, the rate of peripheral neuropathy did not differ between the two groups (4). In the Fermantle Diabetes Study (16), the prevalence of retinopathy was increased twofold among GADA positive patients as compared to negative patients. However, several other studies have found comparable rates of retinopathy and nephropathy among GAD65 positive and negative patients (17–19). In the most recent study and to date the only large prospective trial (3), the incidence of microvascular disease did not differ between GADA positive and negative patients. In addition, the rate of progression to macrovascular events was similar in both groups. In regards to peripheral neuropathy, one study actually found fewer features of neuropathy among GADA positive patients (20). Our study specifically examined the relationship between gastroparesis (a form of autonomic neuropathy) and autoantibody positivity. We found no differences in rates of delayed gastric emptying, or in percent retained at either 2 or 4 h among the GADA positive and GADA negative phenotypically T2DM patients. In addition, there were no differences in symptom severity scores (PAGI-SYM) or in the GCSI between the two groups. We also did not find that rates of peripheral neuropathy differed between GADA positive and negative subjects.

A surprising finding of the study was the portion of subjects who apparently were misclassified in regards to their type of diabetes. For the registry, classification of diabetes was obtained from medical records and/or by patient self-report. Twenty five of the 113 subjects with type 1 diabetes had normal and or elevated C-peptide levels. Since individuals in this group had been diagnosed on average 22 years prior to this assessment, one would have anticipated low c-peptide levels. Thus, 22% of individuals who classified themselves and/or were classified by their health-care provider as having type 1 diabetes probably had T2DM. In addition 7 of the 90 subjects (8%) with the diagnosis of T2DM had low C-peptide levels. However, because of the long duration of diabetes (mean 13.4 years) in this group, it is possible that these individuals had higher levels at diagnosis and that the current low C-peptide levels represented the progressive beta-cell loss seen in T2DM making it impossible to determine if they were misclassified. It must be acknowledged that this potential misclassification is a limitation of this and perhaps other studies involving diabetes. We did not perform any genetic testing or family history to help make the diagnosis of maturity-onset diabetes of the young (MODY). MODY is a form of diabetes classically presents as non-insulin-requiring diabetes in lean individuals typically younger than 25 with evidence of autosomal dominant inheritance. MODY accounts about 1% of all cases of diabetes mellitus (21).

In conclusion, GAD65 antibodies were present in 13% of our phenotypic T2DM patients with symptoms of gastroparesis suggesting presence of LADA. GADA positivity in phenotypic T2DM patients did not associate with delayed gastric emptying or gastroparesis symptom severity. Low C-peptide was associated with insulin use. Some patients labeled as T1DM had normal C-peptide levels suggesting a misclassification. Both the C-peptide levels and GADA positivity could be helpful in correct classification of diabetes.

## Ethics Statement

The Gastroparesis Clinical Research Consortium (GpCRC) Gastroparesis Registry protocol and consents were approved by the GpCRC Steering Committee and by the NIDDK-appointed Data and Safety Monitoring Board. Also, they were reviewed and approved by each institution’s IRB, including the Data Coordinating Center’s IRB. All subjects gave written informed consent in accordance with the Declaration of Helsinki.

## Author Contributions

ES: data interpretation and writing and revising manuscript. CH: study conceptualization, data interpretation, and writing manuscript. LW and PM: statistical analysis, data interpretation, and writing manuscript. AR and JCalles: data interpretation and revising manuscript. GF and FH: revising manuscript. WH, KK, LN, WS, TA, IS, RM, PP, and JClarke: patient recruitment and revising manuscript. JT: statistical analysis, data interpretation, and revising manuscript. HP: study conceptualization, patient recruitment, data interpretation, and writing manuscript.

## Conflict of Interest Statement

The authors declare that the research was conducted in the absence of any commercial or financial relationships that could be construed as a potential conflict of interest.
